# Modulation of Interhemispheric Inhibition between Primary Motor Cortices Induced by Manual Motor Imitation: A Transcranial Magnetic Stimulation Study

**DOI:** 10.3390/brainsci11020266

**Published:** 2021-02-19

**Authors:** Dongting Tian, Shin-ichi Izumi, Eizaburo Suzuki

**Affiliations:** 1 Department of Physical Medicine and Rehabilitation, Tohoku University Graduate School of Medicine, Sendai 980-8575, Japan; izumis@med.tohoku.ac.jp (S.-i.I.); esuzuki@yachts.ac.jp (E.S.); 2 Department of Physical Medicine and Rehabilitation, Tohoku University Graduate School of Biomedical Engineering, Sendai 980-8575, Japan; 3 Department of Physical Therapy, Yamagata Prefectural University of Health Sciences, 260 Kamiyanagi, Yamagata 990-2212, Japan

**Keywords:** transcranial magnetic stimulation, interhemispheric inhibition, imitation, mirror neuron system, ipsilateral silent period

## Abstract

Imitation has been proven effective in motor development and neurorehabilitation. However, the relationship between imitation and interhemispheric inhibition (IHI) remains unclear. Transcranial magnetic stimulation (TMS) can be used to investigate IHI. In this study, the modification effects of IHI resulting from mirror neuron system (MNS) activation during different imitations are addressed. We measured IHI between homologous primary motor cortex (M1) by analyzing the ipsilateral silent period (iSP) evoked by single-pulse focal TMS during imitation and analyzed the respective IHI modulation during and after different patterns of imitation. Our main results showed that throughout anatomical imitation, significant time-course changes of iSP duration through the experiment were observed in both directions. iSP duration declined from the pre-imitation time point to the post-imitation time point and did not return to baseline after 30 min rest. We also observed significant iSP reduction from the right hemisphere to the left hemisphere during anatomical and specular imitation, compared with non-imitative movement. Our findings indicate that using anatomical imitation in action observation and execution therapy promotes functional recovery in neurorehabilitation by regulating IHI.

## 1. Introduction

Interhemispheric inhibition (IHI) is commonly referred to as the physiological process of information transfer between the two hemispheres of the human brain [1], functioning as a mediator of bilateral coordination [2,3]. Since the last century, numerous studies have examined IHI using behavioral experiments [4,5] and assorted noninvasive brain stimulation (NIBS) techniques [6,7,8,9]. In 1992, Ferbert et al. [10] have first proven the existence of IHI between primary motor cortices (M1–M1 IHI) using paired-pulse TMS and established the interhemispheric competition model. Moreover, the interhemispheric facilitation effect was also observed in the paired-pulse TMS paradigm [11]. Nevertheless, apart from paired-pulse TMS, the single-pulse TMS paradigm can also be used for assessing IHI [12]. The ipsilateral silent period (iSP), defined as the transient electromyographic (EMG) activity interruption evoked by focal TMS of the ipsilateral primary motor cortex (M1) in a contracting muscle, could measure M1–M1 IHI directly as a complementary measurement to the paired-pulse TMS paradigm [13,14].

Recently, opinions about interhemispheric control of unilateral movements have come into sight (see [15]). During a unilateral movement, IHI inhibiting the static hemisphere increased, functioning to suppress mirror movements [16,17]. Alternatively, Liepert et al. [18] have suggested that higher bilateral motor performance was related to less inhibition between the hemispheres among healthy populations. Thus, a stronger intensity of IHI leads to fewer mirror activities in unilateral movements but weakens the performance of bilateral coordination. From this perspective, there exists a counterbalance between IHI intensity and performance of unilateral and bilateral movements. However, under the influence of brain lateralization (i.e., hemispheric dominance), during bimanual movements, IHI tends to manifest an asymmetric pattern in healthy people. Among right-handed individuals performing bimanual isometric contraction, IHI tends to show an asymmetric tendency benefiting the dominant hemisphere (i.e., the left hemisphere), whereas, in left-handed people, the asymmetry is not as clear; IHI from both hemispheres was observed to be more equalized in left-handed individuals than that in right-handed people [19].

The original balance of IHI was interrupted by monohemispheric stroke, demonstrating that the intact hemisphere excessively inhibits the damaged hemisphere [20,21,22,23,24]. This imbalance was considered correlated with the poor motor performance [20,21,25]. However, in a one-year longitudinal study, Xu J et al. [26] have reported that during post-stroke recovery, the imbalance of IHI became even greater over time as motor function recovers; therefore, they cast doubt on the previously considered positive correlation between the balance of IHI and motor recovery. Apart from the interhemispheric competition model, a new explanation, known as the bimodal-balance recovery hypothesis [27], proposed that IHI from the contralesional motor cortex to the ipsilesional motor cortex can vary according to the amount of ipsilesional neural reserve and pathways available for recovery. In patients with mild upper limb motor impairment, inhibition intensity targeting the damaged hemisphere was proven to be positively related to impairment severity, hindering function recovery. Alternatively, in patients with severe impairment when little ipsilesional neural structures were reserved, IHI targeting the damaged hemisphere decreased as the motor function worsened, by which the reduced IHI was considered beneficial for recovery. This hypothesis was supported by a subsequent study [28], which has defined a criterion for stratifying the impairment to be upper extremity Fugl–Meyer (UEFM) score 43 in patients with chronic stroke. IHI reduction targeting the damaged hemisphere (i.e., IHI rebalance) in patients with severe stroke was thought to facilitate motor recovery. In summary, despite different theories concerning the topic, consensus on neural mechanisms of IHI balance regarding motor function remains unclear and demands further investigation.

Among the strategies applied to post-stroke rehabilitation, action observation and action execution (AO/AE) therapy were considered an effective method to promote motor recovery [29,30,31,32]. The first report supporting the effectiveness of AO/AE therapy was published in 2007 [29], suggesting that apart from repetitive training, motor recovery can be furtherly enhanced by concurrent AO. Moreover, neuroimaging studies have identified not only the excitation of motor-related cortical areas during AO but also a part of the inferior parietal lobule, inferior frontal gyrus and ventral premotor cortex, known as the core of the mirror neuron system (MNS) [33,34,35]. The MNS was first discovered in macaques [36] and then was proven to have the same existence in the human brain, mainly located in the frontoparietal areas [37,38]. Other than AO, the MNS was also observed to be activated during action recognition [39] and AE [40], motor preparation [41], imitation [42,43], language [44] and emotional recognition (e.g., empathy) [43,45]. The activation of the MNS is a possible neural substrate of AO therapy, being a reason for better post-stroke recovery compared with regular rehabilitative training [29,30].

Imitation, as the integration of AO/AE, was proven to involve the sensorimotor cortex, supplementary motor area (SMA) and cerebellum more than observation or motor imagination alone [46,47]. Traditionally, the concept of “motor imitation” consists of two patterns: anatomical imitation (AI, or non-mirror imitation) and specular imitation (SI, or mirror-image imitation). In AI, the imitator’s left side corresponds to the demonstrator’s left side. However, in SI, the imitator’s right side corresponds to the demonstrator’s left side. SI is more spontaneous than AI among children and adults [48], which is possible because mirror neurons are mainly located in the frontoparietal cortex, which is triggered more easily when performing mirror-image actions [49,50]. In terms of cerebral activation, AI, although more difficult and less spontaneous than SI, is proven to demonstrate a wider and greater cerebral excitation of both hemispheres than SI [50,51]. Thus, despite the different involvement of the MNS between AI and SI, motor imitation, as a subset form of voluntary movement, relies on the integration of both the MNS and motor-related cortices (i.e., sensorimotor cortex, SMA and premotor cortex) with the latter also modulating IHI during voluntary movements.

Different from crossed innervation of motor cortices, the MNS shows a propensity of bilateral (specifically more ipsilateral activation compared with motor cortices) activation during unilateral imitation [52]. A functional magnetic resonance imaging (fMRI) study has reported that despite different involvement of M1, SMA and premotor cortex contralateral to the moving hand, during unimanual imitation, the network of bilateral pars opercularis, bilateral extrastriate areas and bilateral inferior frontal and rostral inferior parietal cortices was significantly activated among healthy participants, with more activation presenting in the hemisphere ipsilateral to the moving hand [51,52]. Moreover, in terms of stroke patients, bilateral activation of the MNS areas induced by mirror visual feedback therapy was considered to be related to visuomotor remapping and remodulation of ipsilesional M1 [53,54].

However, whether the involvement of the MNS can directly modulate IHI between homologous primary motor cortices during unilateral voluntary movement is unclear. In other words, compared with common voluntary movement, when performing imitation movements involving the MNS, the change in the intensity or balance of M1–M1 IHI due to MNS involvement has not been systematically investigated yet. Moreover, whether imitation patterns (i.e., AI and SI) and handedness (i.e., hemisphere dominance), which demand different participation of the MNS and other areas, affect M1–M1 IHI differently from each other remains doubtful.

In this study, we addressed the modification effects of M1–M1 IHI induced by the activation of the MNS during different imitation patterns among healthy right-handed adults. Moreover, we examined the influences of hand dominance (hemispheric dominance) during unimanual imitation on IHI. For these purposes, we designed a series of experiments based on single-pulse TMS. We measured IHI between homologous M1 using iSP evoked by single-pulse focal TMS during imitation of intransitive movements and analyzed the respective IHI modulation and chorological plastic change under different experimental conditions (imitation patterns).

Considering the results reported in prior studies, we made the following hypotheses:

(1) Imitation induces greater disinhibition of bilateral IHI than voluntary movement alone. However, the IHI balance may remain unchanged during imitation due to congruent bilateral IHI reduction, as previously reported in paired-pulse TMS studies [55,56,57].

(2) Compared with SI, AI can activate more the MNS and bilateral motor network and, therefore, can further reduce IHI in both directions.

(3) Imitation using the dominant hand (in this context, the right hand) will result in changes in the IHI balance toward inhibiting the nondominant hemisphere. Reversely, imitation using the nondominant hand (in this context, left hand) will tip the IHI balance toward inhibiting the dominant hemisphere, yet the degree of balance change will be smaller than that when performing imitation using the dominant hand.

IHI has been examined using numerous approaches since the last century, yet the neural mechanisms of interhemispheric interaction in voluntary movements still demand further investigation. By virtue of novel measuring technologies nowadays (e.g., fMRI and NIBS), identifying the neural substrates and information transit process between hemispheres under different task conditions becomes possible and is of great interest. However, although TMS, as a subset of NIBS, was expressed as “noninvasive”, in actuality, TMS can result in safety issues if inappropriate care and safety caution was taken. Despite low incidence in healthy populations, side effects reported for the use of TMS or repetitive TMS (rTMS) include TMS-provoked seizures, hearing safety issues, cognitive effects or potential susceptibility of cerebral dysfunction [58,59]. Therefore, if IHI balance and brain excitability can be modulated by MNS activation, therapies based on MNS activation may become a substitute of TMS (or rTMS) to moderate IHI when there are contraindications for the patient to receive TMS or rTMS treatment (e.g., intracranial ferromagnetic metal implants, or a history of epilepsy). With the advantages of self-training viability and capability to enhance cerebral excitability in a more natural way, incorporating MNS activation in neurorehabilitation may rebalance IHI with fewer adverse effects than NIBS. Moreover, unveiling the basic mechanisms underlying IHI and MNS activation in voluntary motor tasks helps deepen the current understanding of IHI and, thus, may help develop novel rehabilitation therapies based on these mechanisms, such as the combination of motor imitation and NIBS technology, to modify IHI balance effectively. Ultimately, being a basic study to explore the interaction of the MNS and IHI in healthy participants, the results of this study may act as the foundation of subsequent studies on IHI and MNS integration in patients with neurological disorders that impaired the cerebral physiology and interhemispheric interactions such as stroke and traumatic brain injuries.

## 2. Materials and Methods

### 2.1. Participants

Thirty healthy right-handed volunteers (10 males; aged 25.1 ± 1.62 years; age range, 23–28 years) were recruited, all of whom gave written informed consent before the study. One participant withdrew from the experiment before the beginning of data collection due to sudden health conditions. The data of the remaining 29 participants were analyzed and reported.

All participants had normal or corrected-to-normal visual acuity. Handedness was confirmed using the Edinburgh Handedness Inventory [60]. No participants reported psychiatric or neurological histories or medication use. Screening for TMS contraindications was performed according to the TMS Adult Safety Screen [61]. The experiment protocol was approved by the Ethics Committee of Tohoku University Graduate School of Medicine (Protocol Identification Number: 2020-1-642) and was conducted in agreement with legal requirements and the Declaration of Helsinki.

### 2.2. Experimental Design

#### 2.2.1. General Design

**Figure 1:**
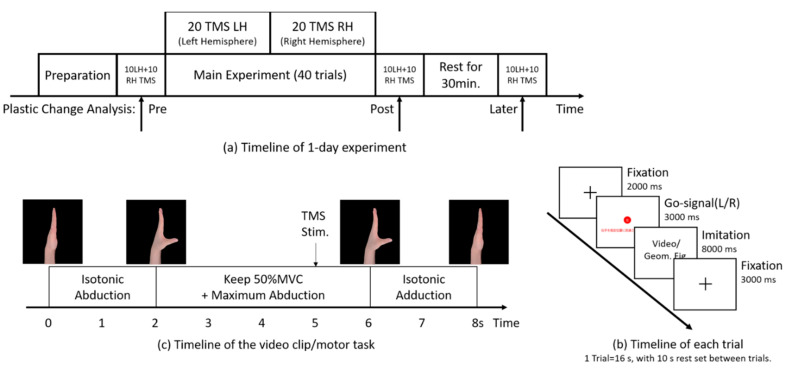
Timeline for the experimental conditions. (**a**) Timeline of 1-day experiment. The main experiment consisted of 40 trials of imitation. Single-pulse transcranial magnetic stimulation (TMS) was delivered to either hemisphere (LH = left hemisphere; RH = right hemisphere) during imitation in each trial. TMS-evoked ipsilateral silent period (iSP) was recorded from the abductor pollicis brevis (APB) ipsilateral to the TMS pulse. Plasticity change was assessed by 60 iSP measurements set immediately before the main experiment (pre), immediately after the main experiment (post) and 30 min rest after the main experiment (later), with 10 iSPs from each hand, measured at each time point. (**b**) Timeline of each trial (16 s). Then, a 10 s rest interval was set between trials. (**c**) Timeline of the 8 s video clip. The video consisted of 2 s isotonic thumb abduction, 4 s retention at the maximum abduction position with 50% MVC and isotonic thumb adduction for 2 s. Focal TMS pulse over M1 was delivered at the 5 s time point of the video clip. The pace of each trial was controlled by a 1 Hz metronome synchronized with the 16 s trial.

Based on the aforementioned purposes and hypotheses, we designed three main experimental conditions in this study: AI condition, SI condition and non-imitative (NI) movement (NI condition). Twenty-nine participants underwent the three main experimental conditions in a random order for 3 consecutive days without further grouping. The experiment on each day consisted of the preparation, main experiment and plastic change analysis. The timeline of the 1-day experiment is demonstrated in Figure 1a.

In the preparation phase, clarification of the experiment procedures, EMG measurements and TMS basic assessment were performed to ensure optimal prerequisites for the upcoming main experiment and plastic change analysis.

During the main experiment phase, the participants performed 40 trials in total (each trial lasts for 16 s with a 30 s interval; the timeline is shown in Figure 1b), with 20 single-pulse TMS delivered to the left and right hemispheres each (left/right in random order). TMS was delivered to the optimal abductor pollicis brevis (APB) representational area in M1 (M1–APB representation hotspot) at the 5 s time point of the video clip (the timeline of the video clip is shown in Figure 1c) during stable isometric thumb abduction of 50% maximum voluntary contraction (MVC), and iSP during imitation was recorded from the APB [62] ipsilateral to the TMS pulse.

Following the main experiment, plasticity change analysis was conducted by comparing the change in IHI assessed by 60 additional iSP measurements (apart from the main experiment) set at three time points—immediately before the main experiment (Pre), immediately after the main experiment (post) and 30-min rest after the main experiment (later)—to examine the plastic effects of imitation on IHI.

#### 2.2.2. Preparation

All experiments were conducted in a well-lit quiet laboratory. The participants were comfortably seated on a chair facing a computer monitor 100 cm away from the front, with both their arms placed near the trunk (shoulder abduction < 5°) and their forearms rested on the desk.

Before the main experiment, EMG activities of the left and right APB were recorded from the participants. The participants were provided with real-time visual EMG feedback (root-mean-square of real-time raw EMG by 3 s, shown as a moving black line on the screen) while following the instruction to abduct their thumb (left or right, respectively) with different strengths. The EMG output of MVC was calculated from three maximal contractions of the thumb abduction average [63]. After the calculation of MVC, the participants were instructed to abduct their thumbs and maintain the EMG output within the designated range of 50% ± 10% MVC displayed as a green area on the screen [63]. Moreover, to ensure the output of the APB during imitation without visual feedback, we converted the visual feedback into proprioceptive feedback by adopting a spring device (covering 0% MVC to 100% MVC of the APB; details stated in *EMG Feedback Alternation*) for the participants to press with their thumb interphalangeal (IP) joint while receiving concurrent visual feedback on 50% MVC. The experimenter then fixed the position of the spring device for proprioceptive feedback alternation.

Upon completion of the EMG procedures, TMS was delivered to both hemispheres, respectively, to locate the M1−APB representation hotspot and determine the participants’ resting motor threshold (rMT), by which the TMS intensity in the main experiment and plastic change analysis was specified (details stated in *TMS Parameters*).

After EMG and TMS preparation, the participants were instructed on the details of each trial (including the video clip) and all movement elements involved in the main experiment. Moreover, several practice trials without TMS were performed to ensure the participants’ full understanding of the experimental procedures.

#### 2.2.3. EMG Feedback Alternation

**Figure 2:**
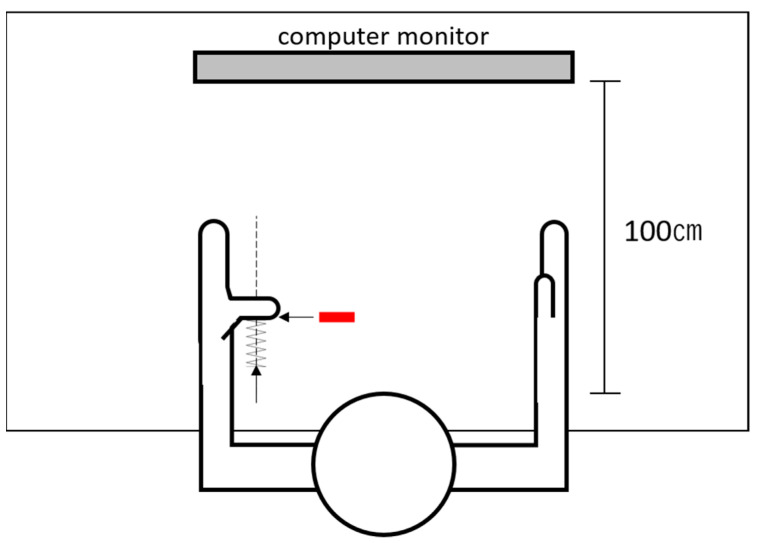
Schematic diagram of the spring device (the left hand as an example). The experimenter pushed the spring device along the vertical trajectory (the dotted line), passing the thumb interphalangeal joint, gradually applying force against the participant’s fully abducted thumb. If the stable electromyographic recording of 50% ± 10% maximum voluntary conTable 50. ± 10% MVC output of the bilateral APB. After the participants managed to maintain 50% ± 10% MVC with visual feedback, feedback alternation procedures were performed subsequently. When the participants reached maximum thumb abduction without force output, the experimenter pushed the spring device along the vertical trajectory passing the thumb IP joint, gradually applying force against the participants’ fully abducted thumb. The participants were instructed to maintain maximum abduction while the spring force was gradually applied to the thumb IP joint. If stable EMG recording of 50% ± 10% MVC appeared on the screen, the bottom of the spring device was then fixed on the desk, and a red sign was attached to the desk at the place that was covered unseen by the participants beneath their thumb. In the main experiment, the participants were instructed to ‘abduct the thumb fully and press against the spring device, covering the red sign to keep the optimal muscle output’ to maintain 50% MVC muscle output when necessary.

Due to the necessity to focus on the video clip in the main experiment, receiving visual feedback of their muscle activities while imitating the movements was impossible to attain for the participants. Considering the report by Baweja et al. [64], during constant isometric contraction at a relatively low force level (i.e., 50% MVC), healthy volunteers could maintain muscle activity without significant high force error after the removal of visual feedback. As an inference, we designed a spring-based pressing device (here referred to as ‘spring device’) to convert visual feedback to proprioceptive force feedback provided by the spring during thumb abduction (Figure 2).

With both visual and proprioceptive feedback, the participants performed five practice trials maintaining 50% MVC output (each lasts 10 s, with a 10 s rest interval set between trials). Then, the visual feedback was removed, and the participants underwent the same practice trials only with proprioceptive feedback provided by the spring device. The participants proceeded to the main experiment if they managed to maintain the output in the designated range without visual feedback for five consecutive practice trials for each hand.

The procedures of feedback alternation were conducted to the participants’ both hands, respectively, and two spring devices for the left and right hands were used in the experiment. Despite the feedback alternation, the recordings from both APB were monitored by the experimenter throughout the experiment. If the EMG deviated out of 50% ± 10% MVC, the participants were orally instructed by the experimenter to adjust their abduction output accordingly.

#### 2.2.4. Main Experiment

In the main experiment, the participants were instructed to observe a unimanual thumb abduction–adduction video clip and imitate simultaneously for 40 trials (20 trials of left-hand movements and 20 trials of right-hand movements in total) per day. Each trial (the timeline is shown in Figure 1b) lasted for 16 s, with an 8 s video clip for imitation embedded. First, a black fixation cross was displayed on the screen for 2 s, followed by a “go” signal consisting of a color-filled circle (red or cyan lasting for 3 s) indicating the side (left or right) of the imitation hand in the video clip for the participants to imitate, which was randomized in each trial, and a corresponding literal instruction guiding the participants to abduct the non-imitation thumb to reach 50% MVC before the video clip. Next, the 8 s video clip (the timeline is shown in Figure 1c) was displayed. While observing the video clip, the participants should imitate the movements in the video simultaneously and keep 50% MVC of thumb abduction by pressing the spring device while imitating maximum thumb abduction. The video clip was then followed by a 3 s continuous fixation period to prevent possible attention fluctuation after imitation. The pace throughout the trials was controlled using a 1 Hz metronome synchronized with the 16 s trial. Single-pulse TMS was delivered over the left M1–APB representation hotspot in 20 trials and 20 trials over the hotspot of the right M1 (left/right in random order), at the 5 s time point in the video clip where 50% MVC isometric contraction of the APB was consistently maintained. TMS–evoked iSP in the main experiment was recorded from the APB ipsilateral to the magnetic stimulation.

In 3 consecutive days, the participants performed three imitation patterns referred to as AI, SI and NI movement in random order. In AI, participants observed the displayed video and corresponded their left side to the demonstrator’s left side (and vice versa) in the video. However, in SI, participants observed the video and corresponded their left side to the demonstrator’s right side (and vice versa). In NI condition, the movement video was replaced by a series of four meaningless geometric figures for 8 s (each figure appeared for 2 s), and the participants performed the movements same as the phase shown in Figure 1c while observing the geometric figures.

The video clips displayed in AI and SI were the same series of unimanual movements of both hands, precisely following the phase shown in Figure 1c. To avoid unnecessary brain activity caused by judging the moving side after the video clip began, the side of the hand moving in the video clip was indicated by the filled circle in a different color preceding the video (red for the left hand and cyan for the right hand) in each trial. In the AI condition, the participants were instructed to use their hand contralateral to the side indicated by the filled circle. However, in the SI condition, the participants used their hand ipsilateral to the side indicated. In the NI condition, the participants also performed unimanual movements according to the color of the circle. For each trial, the side of the imitation hand was randomized with 10 left and 10 right hands moving in the video clip among the 20 trials where TMS was delivered to one hemisphere. During the 3 s appearance of the color-filled circle, the participants should fully relax their hand for imitation (or the hand performing the movement in the NI condition) and abduct the thumb of the other hand to keep maximum abduction with 50% MVC until the end of the trial, as a preparation of the upcoming movement and TMS measurement.

#### 2.2.5. Plastic Change Analysis

Plastic change analysis was performed by comparing the change in IHI over time measured using 20 iSP measurements (10 iSPs from each hemisphere) at each time point (pre, post and later in Figure 1a, with inter-stimulation rest of 5 s to prevent possible fatigue). iSP duration acquired from pre time point measurement in the plasticity change analysis was the baseline value of iSP in each experiment. In the plastic change analysis, we adopted the same iSP measurement parameters as that in the main experiment (but without abduction of the hand contralateral to TMS pulse) and analyzed the change in iSP throughout the experiment.

#### 2.2.6. EMG Recording

EMG activities were recorded from the APB using disposable surface electrodes (Ambu Blue Sensor N, N-00-S/25, Ambu A/S, Ballerup, Denmark) placed over the APB muscle belly (belly–belly montage) and ulnar styloid process (reference electrodes) of both hands. EMG raw signal was amplified 1000×, band-pass filtered (20–450 Hz) with a sampling rate of 10 kHz and stored on a computer for offline analysis. The EMG time zone for analysis was set to 8 s from 5 s before TMS to 3 s after TMS (i.e., the same duration as the displayed video clip). EMG traces that deviated out of 50% ± 10% MVC from 500 ms before TMS or traces with unwanted muscle activities were discarded from further analyses.

#### 2.2.7. TMS Parameters

Single-pulse TMS was delivered to either hemisphere using a figure-of-eight coil (70 mm external diameter of each loop) connected to a Magstim-200^2^ magnetic stimulator (Magstim200^2^, Magstim Co., Ltd., Whitland, UK) with a monophasic current waveform. The M1–APB representation hotspot of both hemispheres were marked using a pen, where slightly suprathreshold TMS elicited the largest and most consistent motor-evoked potentials (MEPs) in the APB muscle. The junction center of the coil was placed tangentially to the participants’ scalp by an articulated mechanical arm (Manfrotto 244, VitecGroup, Italy) at the M1–APB representation hotspot. The handle of the coil was oriented backward and at an angle of 45° from the midsagittal line over M1 [65]. With the usage of a chin-head rest, the position of the participant’s head can therefore be fixed for precise delivery of the TMS pulse.

The rMT of each hemisphere was determined before the experiment according to the report by Chen et al. [66], as the TMS intensity (percentage of the maximum stimulator output (MSO)) that produced five MEPs (≥50 μV peak-to-peak amplitude) of 10 consecutive stimuli from the APB.

TMS pulse was synchronized with the video clip using custom MATLAB program scripts executed in MATLAB 2019b (The MathWorks, Inc., Natick, MA, USA) and delivered to the M1–APB representation hotspot precisely at the 5 s time point of the video clip. The intensity of focal TMS was set to 120% rMT [17,19,67] of the corresponding hemisphere.

### 2.3. Outcome Measures

Raw EMG data were processed using LabChart 8 (AD Instruments, Sydney, Australia). Pre-stimulus EMG was calculated from the averaged EMG 500 ms before TMS. The iSP onset was defined as the time point at which EMG fell below 75% of the mean pre-stimulus EMG for more than 5 ms in the time window of 30–60 ms after the stimulus [68]. The offset of iSP was defined as the point at which the EMG returned more than 75% of the mean pre-stimulus EMG [69,70]. Trials without observable iSP (at least 25% of EMG suppression lasting less than 10 ms) were excluded before averaging the EMG activity for further analysis. The duration of iSP was calculated in milliseconds as the time course between iSP onset and offset.

Data recorded in each day (condition) for one participant was processed as follows: First, trials with unwanted EMG activities or no observable iSP were excluded. Second, the valid EMG recordings measured in each direction and epoch (LH–RH/RH-LH in dominant hand (DH) imitation/nondominant hand (NDH) imitation/pre/post/later) were averaged separately for further analysis. Then, a custom-made MATLAB R2020b script for iSP automatic calculation was run to calculate the duration of iSP in the averaged EMG of each direction and epoch. The automatically calculated iSP duration (in milliseconds) was then saved in our database for further statistical analyses.

iSP duration was considered the parameter indicating IHI in this study, and thus, the iSP durations of both directions were recorded for statistical analysis. Meanwhile, the side of the imitation hand (DH and NDH) were also labeled with the iSP recording in each trial to analyze the effect of hand dominance on IHI. All iSP duration outcomes throughout the experiment were separated into iSP in both directions (the left hemisphere to the right hemisphere as LH–RH, and the right hemisphere to the left hemisphere as RH−LH). Moreover, we adopted a ratio named IHI asymmetry ratio (IAR) [71] to depict the balance of bidirectional IHI in the main experiment and plastic change analysis. IAR was calculated as follows:

IAR = iSP_LH–RH/_iSP_RH–LH_ (1)

An IAR of 1 indicated that the IHI of both directions were identical and in equilibrium. An IAR > 1 indicated that IHI from LH–RH was greater than the opposite direction. Conversely, an IAR < 1 indicated that IHI from RH–LH was greater than that from LH–RH. The baseline IAR of each participant was calculated from baseline iSP values. Additionally, at each time point in the plastic change analysis (pre, post and later), an average of the 10 measured iSP durations (in LH–RH or RH–LH direction) was computed and recorded as an “average plasticity value (pre/post/later)”. As a result, six averaged plasticity values (three time points × two directions) were acquired in each day with a time order for further analysis of the chronological plastic changes.

### 2.4. Statistical Analysis

The data of all iSP durations measured from the main experiment and plastic change analysis were included in the statistical analysis. The within-subject difference in baseline iSP duration (measured in pre-time point plastic change analysis) between AI, SI and NI conditions were analyzed using one-way analysis of variance (ANOVA) in both directions, respectively.

The variances of iSP and IAR (i.e., ΔiSP LH–RH, ΔiSP RH–LH and ΔIAR) were calculated by subtracting the baseline iSP duration from the iSP durations measured from the main experiment and IAR accordingly. A series of analyses of covariance (ANCOVA) of ΔiSP LH–RH, ΔiSP RH–LH and ΔIAR were performed separately with *Condition* (AI, SI and NI) and *Dominance of imitation hand* (DH and NDH) set as covariables.

The averaged plasticity values from *pre–post–later* plastic change analysis on each day of the experiment were analyzed in separate directions using repeated measures ANOVA (RMANOVA) to reveal the chronological changes in plasticity effects resulting from different experimental conditions.

A post hoc test was performed using Bonferroni’s correction following all statistically significant results or tendencies in the aforementioned analyses. *p* values of < 0.05 were used to denote statistical significance. Statistical analyses were performed using custom MATLAB R2020b scripts and Statistical Package for the Social Sciences version 26.0 for Windows (IBM Corp., Armonk, NY, USA) for double confirmation of the statistical results.

## 3. Results

Twenty-nine healthy adults were tested, all of whom could follow the instructions given and maintain the required intensity of volitional contraction throughout the experiment. The final analysis consisted of 87 days of experiment data collected from 29 participants. No participant reported any discomfort or side effects during the experiment and three days thereafter.

**Figure 3:**
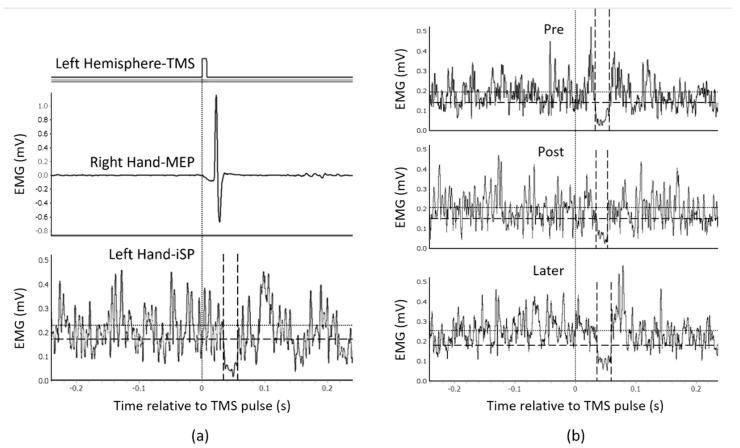
Examples of ipsilateral silent period (iSP) recordings and plastic change analysis. (**a**) An example of iSP measurement (averaged from 10 TMS trials) of a representative participant. TMS was delivered to the left hemisphere. (**b**) Example of a series of plastic change analyses of another representative participant’s EMG data recorded under anatomical imitation condition. The vertical dotted line indicates the time point of the TMS pulse. The horizontal dotted line indicates the average muscle activities 500 ms before TMS, recorded from the APB muscle. The vertical dashed lines indicate the onset/offset of iSP. The horizontal dashed line indicates the threshold for iSP calculation (75% average muscle activities as mentioned in *Outcome Measures*).

Figure 3 illustrates examples of iSP analysis. In example (a), TMS was delivered to the left hemisphere. An obvious MEP was recorded from the right APB after TMS, whereas iSP of the left APB could be measured by analyzing the averaged rectified EMG activity according to the criteria of iSP onset and offset. Example (b) shows a basic process of plastic change analysis. ISP durations measured at the pre, post and later time points (computed in milliseconds) were included in the plastic change analysis, from which the change in iSP through the three time points could be revealed statistically.

An overall summary of the data included in the statistical analysis is shown in Table 1. All iSP durations were analyzed in both directions separately (RH–LH and LH–RH), in association with IAR, indicating interhemispheric balance. In terms of baseline values, no significant within-subject difference in baseline bidirectional iSP duration (*p =* 0.489 in LH–RH; *p =* 0.261 in RH–LH) or IAR (*p =* 0.428) was observed between the three experimental conditions as analyzed using one-way ANOVA. Consequently, the modulation observed in the main experiment afterward and chronological changes could not be attributed to baseline differences.

### 3.1. iSP Modulation during Different Imitation Conditions

The average rMT of the participants was 55.93% ± 7.84% MSO for the LH and 58.10% ± 7.16% MSO for the RH. Accordingly, TMS intensity was set to 67.11% ± 9.41% MSO for the LH and 69.72% ± 8.59% MSO for the RH, as 120% rMT mentioned in TMS parameters.

**Figure 4:**
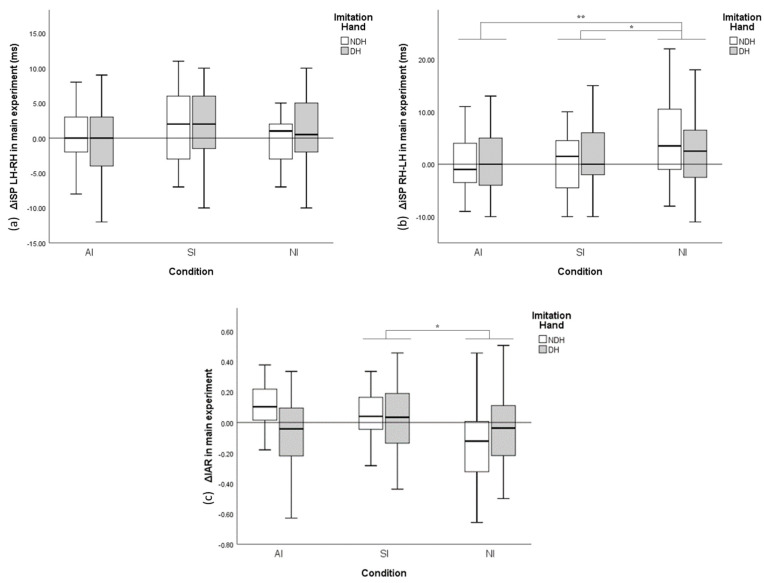
Variance (Δ) of bidirectional ipsilateral silent period (iSP) duration and interhemispheric asymmetry ratio (IAR) compared with baseline during the main experiment. (**a**) ΔiSP LH–RH with different conditions and imitation hands. (**b**) ΔiSP RH–LH with different conditions and imitation hands. (**c**) ΔIAR with different conditions and imitation hands. The y-axis of A and B display the duration variance of iSP, with positive values indicating iSP (IHI) increase and negative values indicating iSP (IHI) decrease. The y-axis of C indicates the variance of IAR. Error bars represent a 95% confidence interval (CI). * *p* < 0.05; ** *p* < 0.01.

ANCOVA of ΔiSP RH–LH in the main experiment (Figure 4b) revealed a significant effect of *Condition* on ΔiSP duration RH–LH (*p =* 0.003), but not of *Dominance of imitation hand* (*p =* 0.462). The post hoc test revealed significant differences between AI and NI conditions (*p =* 0.006) and between SI and NI conditions (*p =* 0.019). Alternatively, *Condition* (*p =* 0.160) or *Dominance of imitation hand* (*p =* 0.791) had no significant effect on ΔiSP LH–RH ANCOVA (Figure 4a). The variation of IAR (ΔIAR) (Figure 4c) showed that *Condition* (ANCOVA, *p =* 0.021), but not *Dominance of imitation hand*, had a significant effect (*p =* 0.491). The results of the post hoc test revealed a significant difference between SI and NI conditions (*p =* 0.019).

### 3.2. Chronological Plastic Changes of iSP

**Figure 5:**
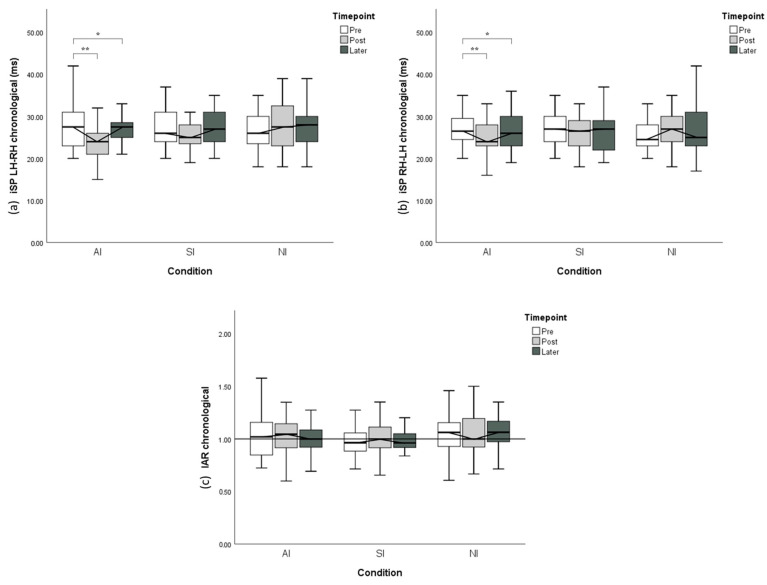
Chronological variation of the ipsilateral silent period (iSP) from plastic change analysis. (**a**) iSP LH–RH with different conditions and time points. (**b**) iSP RH-LH with different conditions and time points. (**c**) Interhemispheric asymmetry ratio IAR with different conditions and time points. The y-axis of A and B displays iSP duration, with shorter iSP duration indicating less interhemispheric inhibition. The y-axis of C indicates IAR computed from the corresponding iSP. Error bars represent 95% CI. * *p* < 0.05; ** *p* < 0.01.

RMANOVA with the independent variable of *Time* (pre, post and later) and *iSP duration* as a dependent variable was performed independently considering iSP directions and IAR (Figure 5).

Under the AI condition, RMANOVA indicated that significant time-course changes through the experiment were observed in both LH–RH (*p* < 0.001) and RH–LH (*p =* 0.001) directions. The post hoc test of iSP LH–RH revealed that iSP duration was significantly reduced from the Pre time point to the post time point (*p* < 0.001) and did not return to baseline 30 min after the imitation (*p =* 0.03). Similarly, the post hoc test of iSP RH–LH revealed a significant decline in iSP duration from the pre time point to the post time point (*p =* 0.001) and persistent iSP reduction 30 min afterward (*p =* 0.025). However, no significant chronological change was revealed regarding IAR in the AI condition (*p =* 0.396).

In the SI condition, despite the same modulation pattern through time as that in the AI condition, no significant time-course changes in iSP were observed in LH–RH (*p =* 0.65), RH–LH (*p =* 0.68) or IAR (*p =* 0.41).

In the NI condition, no significant chronological iSP changes in LH–RH (*p =* 0.58), RH–LH (*p =* 0.40) or IAR (*p =* 0.99) were found.

## 4. Discussion

In this study, we seek to address the modification effects of M1–M1 IHI resulting from MNS activation by examining the modulation of IHI through three experimental conditions (AI, SI and NI movement). We used the single-pulse TMS (i.e., iSP) paradigm to assess IHI between M1 dynamically during voluntary movement of right-handed adult participants. The results of the experiments indicated that the effects of imitation on IHI were significantly different from those of NI movement, which were demonstrated both during the process of imitation and the chronological change results after the imitation. However, we found no significant modulation in M1–M1 IHI for SI to differ from NI movement in terms of chronological plastic effects. Based on the evidence of the intrinsic difference between imitation (with MNS activation) and NI movements (without MNS activation) proven by various techniques [72,73,74], this study provides the first evidence supporting the hypothesis that imitation (specifically AI) can induce greater disinhibition on IHI between M1 than voluntary movements alone. Furthermore, we found that AI can modulate M1–M1 IHI more effectively than SI, and the plastic effects of AI can last for a period after the imitation per se.

### 4.1. M1–M1 IHI Modulation during Imitation

As abundantly discussed in several studies, an obvious difference exists between AI and SI despite the common involvement of the MNS regarding imitation patterns, developmental spontaneity and cerebral activation [75,76]. Moreover, infants develop motor skills by imitating others’ movements, and such imitation spontaneously follows the patterns of SI [48,77]. Moreover, SI is found to be a more natural (default) pattern of simple-task imitation among adults, as it is considered to be easier to imitate specularly [49,78]. As one of the basic functions of IHI is to suppress mirror movements [15,79,80], the necessity to suppress the propensity to imitate in a specular pattern indicates the involvement of IHI during AI. As a result of this, greater disinhibition effects emerged in anatomical imitation, but not in specular imitation. In this study, we observed different modulation effects on IHI between the AI and SI conditions compared with NI conditions, which was according to the aforementioned evidence.

During imitation, iSP duration resulting from RH–LH IHI declined more in the AI condition than that in the SI condition and that in the SI condition declined more than that in the NI condition. Interestingly, the ratio of bidirectional IHI (i.e., IAR) during imitation showed a significant difference between SI and NI conditions, but not in AI and NI conditions, indicating that IHI balance may be modulated differently in AI and SI. Moreover, in a recent study by Pierpaoli C et al. [81], patients who underwent callosotomy tended to imitate specularly in both free (66%) and instructed conditions (61%), producing the implication that AI involves more callosal function and interhemispheric interaction. These results conform to those of previous studies suggesting more cerebral activation in AI [50,51,76] and the hypothesis that AI can modulate IHI more efficiently due to more involvement of the MNS and bilateral motor network.

However, no modulation effect was observed on LH–RH IHI between the three conditions in this study. A possible reason for this result can be expressed by previous findings that IHI from the dominant hemisphere to the nondominant hemisphere in the right-handed population was differently regulated during voluntary movements [82]. Before the onset of dominant (right) hand movement, IHI inhibiting the dominant (left) hemisphere reduced, whereas inhibition targeting the nondominant hemisphere remained still. Interestingly, the disinhibition effect was not observed during nondominant hand movements, in which the interhemispheric balance remained constant before the movement onset [83]. Moreover, during left-hand movements, the dominant left M1 (ipsilateral to the left hand) appeared to be more active compared with the activity of the nondominant right M1 during right-hand movements, which further confirmed the asymmetry of IHI modulation during voluntary movements [67]. Consequently, IHI from the nondominant hemisphere to the dominant hemisphere may be more prone to be affected by voluntary movements compared with the reverse direction among the right-handed population and, thus, can be a potential factor accounting for the negative results of IHI inhibiting the nondominant hemisphere between the three conditions.

### 4.2. Hand Dominance Did Not Affect IHI during Imitation

Contrary to the evidence from other studies in this area that hand dominance affects IHI in unilateral movements [80,82] and our hypothesis, we did not find a significant difference in IHI modulation between dominant-hand and nondominant-hand imitation. Alternatively, it is possible that we failed to identify significant effects due to the movement we adopted in the experimental design. As mentioned in *Materials and Methods*, the movement for imitation was a series of easy, intransitive movements of thumb abduction and adduction. Among right-handed individuals, significant differences in motor performance and cerebral activation between the dominant and nondominant hands usually emerge from complex unilateral movements [84,85,86], and thus, movements in this study may have little impact on IHI modulation in terms of hand dominance. Alternatively, in a paired-TMS study focusing on homologous muscle representations in unimanual sustained contractions, IHI bidirectionally reduced during unilateral isometric contraction compared with the rest state, without evidence of hemispheric dominance [87]. This may indicate that there was essentially little IHI modulation by the dominance of the moving hand, which led to the negative results in this study. Furthermore, we are aware that the movements involved in the main experiment were not merely unilateral thumb abduction and adduction of the imitation hand but also prerequisite maintenance of maximum abduction of the contralateral non-imitation hand. Because of this and previous fMRI–dynamic causal modeling study [88] indicating significantly different IHI modulation in unilateral and bilateral movements, we may infer that the effects of hand dominance were possibly weakened due to the background EMG activities of the non-imitation hand in this study.

### 4.3. Plastic Effects Differ between Imitation Patterns

The outcomes of the plastic change analysis confirmed that different plastic effects of IHI from imitation exist compared with NI movements. Our hypothesis that AI reduces IHI more in both directions than SI was proven by the presence of significant disinhibition effects on bidirectional IHI, which were present immediately and 30 min after AI. To ensure that the effects were caused by imitation rather than baseline difference, we performed a series of one-way ANOVA and found no inter-conditional difference in the baseline iSP duration. Additionally, the disinhibition effects only emerged from AI (but not from SI), which corresponded to the evidence that more of the MNS and motor-related areas are involved in AI than SI [50,51,76].

### 4.4. Significance and Limitation

Based on the outcomes of this study, IHI is modulated during imitation (specifically AI), which differs from that of regular simple voluntary movement. This study provides the first evidence illustrating the relationship between IHI modulation and MNS activation, which can, to a certain degree, account for the underlying neural mechanism of AO/AE therapy applied in clinical neurorehabilitation. Furthermore, since the imitation or imagery patterns of the current AO/AE therapy are not specified in most clinical situations (see [40]), we suggest that using anatomical imitation in neurorehabilitation as a complement to current NIBS technologies can yield better rehabilitation effects, as it may release the degree of IHI following a stroke or traumatic brain injuries and, thus, can promote motor recovery.

Nevertheless, several limitations in this study ought not to be ignored. First, the participants included in this study were all right-handed young adults, and the results from this study may not be applied to left-handed individuals or other age groups, demanding further examination. Second, there was no measurement on rest-state IHI to see the modulation related to the rest state due to the attributes of the iSP paradigm that voluntary muscle contraction is necessary for the measurement. For this reason, further assessing IHI using paired-pulse TMS as a complementary approach for examining IHI modulation in motor imitation is more objective. Third, since the movements adopted in this study were simple intransitive movements, further study regarding transitive, complex movements would be necessary to illustrate the patterns of IHI and MNS activation during imitation. Forth, as iSP measured by single-pulse TMS is considered to reflect IHI mediated by γ-aminobutyric acid B (GABA-B) receptor-mediated cortical interneurons, it demands further investigation to assess the effects that imitation had on IHI using other measurements, for example, paired-pulse TMS paradigm assessing GABA-A-mediated inhibition, or the novel TMS-EEG paradigm measuring interhemispheric signal propagation [89,90]. To sum up, it demands more examination targeting different age groups and hand dominance populations (including neurological patients), different kinds of movement and measuring approaches to unveil the underlying neural mechanisms behind this modulation.

## 5. Conclusions

In conclusion, the results reported in this study provide the first evidence that manual motor imitation, specifically AI, can substantially disinhibit IHI between primary motor cortices (M1–M1 IHI). This study suggests that MNS activation, which can be induced by AO and imitation, modulates M1–M1 IHI more efficiently than regular voluntary movements. As such, our findings, taken together with previous studies, indicate that using anatomical imitation may promote functional recovery in neurorehabilitation by regulating IHI.

## Figures and Tables

**Table 1:** Averaged values of all data analyzed. The standard deviation (SD) of each value was presented in parentheses. LH left hemisphere; RH, right hemisphere; iSP, ipsilateral silent period; IAR, interhemispheric asymmetry ratio; NDH, nondominant hand; DH, dominant hand; Δ, variance compared to baseline.

Averaged iSP Duration (ms)/IAR	Anatomical Imitation			Specular Imitation			No Imitation		
	LH–RH	RH–LH	IAR	LH–RH	RH–LH	IAR	LH–RH	RH–LH	IAR
Pre (baseline)	28.86	27.52	1.07	26.97	27.03	1.01	27.69	25.79	1.09
	(6.46)	(4.50)	(0.25)	(4.33)	(3.51)	(0.17)	(6.80)	(4.02)	(0.27)
NDH imitation	29.59	28.45	1.07	29.07	28.48	1.05	27.83	31.79	0.91
	(6.77)	(7.02)	(0.27)	(5.11)	(6.40)	(0.19)	(6.98)	(8.52)	(0.25)
DH imitation	28.21	28.00	1.03	29.21	28.07	1.06	29.79	30.10	1.03
	(5.92)	(5.11)	(0.27)	(4.85)	(4.72)	(0.22)	(7.97)	(8.92)	(0.27)
Δ NDH imitation	0.72	0.93	0.005	2.10	1.45	0.04	0.14	6.00	–0.18
	(5.13)	(7.26)	(0.30)	(6.12)	(6.39)	(0.18)	(6.20)	(9.41)	(0.39)
Δ DH imitation	–0.65	0.48	–0.03	2.24	1.03	0.05	2.10	4.31	–0.06
	(5.14)	(6.11)	(0.30)	(5.62)	(6.39)	(0.26)	(6.84)	(8.88)	(0.39)
Post	24.76	24.31	1.02	26.38	26.24	1.02	28.52	26.69	1.09
	(5.99)	(4.17)	(0.19)	(5.22)	(4.40)	(0.17)	(7.51)	(4.91)	(0.26)
Later	26.41	26.62	1.00	27.31	26.24	1.06	28.83	27.31	1.09
	(4.97)	(4.16)	(0.17)	(4.36)	(4.48)	(0.22)	(6.35)	(6.47)	(0.26)

## Data Availability

The data presented in this study are available on request from the corresponding author.

## References

[1] Hellige J.B. (1987). Interhemispheric Interaction: Models, Paradigms and Recent Findings. Duality and Unity of the Brain.

[2] Daffertshofer A., Peper C.L.E., Beek P.J. (2005). Stabilization of bimanual coordination due to active interhemispheric inhibition: A dynamical account. Biol. Cybern..

[3] Carson R.G. (2005). Neural pathways mediating bilateral interactions between the upper limbs. Brain Res. Rev..

[4] Marzi C.A. (1999). The Poffenberger paradigm: A first, simple, behavioural tool to study interhemispheric transmission in humans. Brain Res. Bull..

[5] Braun C.M.J., Collin I., Mailloux C. (1997). The “Poffenberger” and “Dimond” paradigms: Interrelated approaches to the study of interhemispheric dynamics?. Brain Cogn..

[6] Bütefisch C.M., Weβling M., Netz J., Seitz R.J., Hömberg V. (2008). Relationship Between Interhemispheric Inhibition and Motor Cortex Excitability in Subacute Stroke Patients. Neurorehabilit. Neural Repair.

[7] Fiori F., Chiappini E., Candidi M., Romei V., Borgomaneri S., Avenanti A. (2017). Long-latency interhemispheric interactions between motor-related areas and the primary motor cortex: A dual site TMS study. Sci. Rep..

[8] Duque J., Hummel F., Celnik P., Murase N., Mazzocchio R., Cohen L.G. (2005). Transcallosal inhibition in chronic subcortical stroke. NeuroImage.

[9] McDonnell M.N., Stinear C.M. (2017). TMS measures of motor cortex function after stroke: A meta-analysis. Brain Stimul..

[10] Ferbert A., Priori A., Rothwell J.C., Day B.L., Colebatch J.G., Marsden C.D. (1992). Interhemispheric inhibition of the human motor cortex. J. Physiol..

[11] Hanajima R., Ugawa Y., Machii K., Mochizuki H., Terao Y., Enomoto H., Furubayashi T., Shiio Y., Uesugi H., Kanazawa I. (2001). Interhemispheric facilitation of the hand motor area in humans. J. Physiol..

[12] Meyer B.-U., Röricht S., Von Einsiedel H.G., Kruggel F., Weindl A. (1995). Inhibitory and excitatory interhemispheric transfers between motor cortical areas in normal humans and patients with abnormalities of the corpus callosum. Brain.

[13] Perez M.A., Cohen L.G. (2009). Interhemispheric inhibition between primary motor cortices: What have we learned?. J. Physiol..

[14] Avanzino L., Teo J.T.H., Rothwell J.C. (2007). Intracortical circuits modulate transcallosal inhibition in humans. J. Physiol..

[15] Beaulé V., Tremblay S., Théoret H. (2012). Interhemispheric Control of Unilateral Movement. Neural Plast..

[16] Vercauteren K., Pleysier T., Van Belle L., Swinnen S.P., Wenderoth N. (2008). Unimanual muscle activation increases interhemispheric inhibition from the active to the resting hemisphere. Neurosci. Lett..

[17] Giovannelli F., Borgheresi A., Balestrieri F., Zaccara G., Viggiano M.P., Cincotta M., Ziemann U. (2009). Modulation of interhemispheric inhibition by volitional motor activity: An ipsilateral silent period study. J. Physiol..

[18] Liepert J., Dettmers C., Terborg C., Weiller C. (2001). Inhibition of ipsilateral motor cortex during phasic generation of low force. Clin. Neurophysiol..

[19] Reid C.S., Serrien D.J. (2012). Handedness and the excitability of cortical inhibitory circuits. Behav. Brain Res..

[20] Duque J., Mazzocchio R., Dambrosia J., Murase N., Olivier E., Cohen L. (2005). Kinematically Specific Interhemispheric Inhibition Operating in the Process of Generation of a Voluntary Movement. Cereb. Cortex.

[21] Murase N., Duque J., Mazzocchio R., Cohen L.G. (2004). Influence of interhemispheric interactions on motor function in chronic stroke. Ann. Neurol..

[22] Kirton A., DeVeber G., Gunraj C., Chen R. (2010). Cortical excitability and interhemispheric inhibition after subcortical pediatric stroke: Plastic organization and effects of rTMS. Clin. Neurophysiol..

[23] Cunningham D.A., Machado A., Janini D., Varnerin N., Bonnett C., Yue G., Jones S., Lowe M., Beall E., Sakaie K. (2015). Assessment of Inter-Hemispheric Imbalance Using Imaging and Noninvasive Brain Stimulation in Patients With Chronic Stroke. Arch. Phys. Med. Rehabil..

[24] Liepert J., Hamzei F., Weiller C. (2000). Motor cortex disinhibition of the unaffected hemisphere after acute stroke. Muscle Nerve.

[25] Ward N.S., Cohen L.G. (2004). Mechanisms Underlying Recovery of Motor Function After Stroke. Arch. Neurol..

[26] Xu J., Branscheidt M., Schambra H., Steiner L., Widmer M., Diedrichsen J., Goldsmith J., Lindquist M., Kitago T., Luft A.R. (2019). Rethinking interhemispheric imbalance as a target for stroke neurorehabilitation. Ann. Neurol..

[27] Di Pino G., Pellegrino G., Assenza G., Capone F., Ferreri F., Formica D., Ranieri F., Tombini M., Ziemann U., Rothwell J.C. (2014). Modulation of brain plasticity in stroke: A novel model for neurorehabilitation. Nat. Rev. Neurol..

[28] Lin Y.-L., Potter-Baker K.A., Cunningham D.A., Li M., Sankarasubramanian V., Lee J., Jones S., Sakaie K., Wang X., Machado A.G. (2020). Stratifying chronic stroke patients based on the influence of contralesional motor cortices: An inter-hemispheric inhibition study. Clin. Neurophysiol..

[29] Ertelt D., Small S., Solodkin A., Dettmers C., McNamara A., Binkofski F., Buccino G. (2007). Action observation has a positive impact on rehabilitation of motor deficits after stroke. NeuroImage.

[30] Celnik P., Webster B., Glasser D.M., Cohen L.G. (2008). Effects of Action Observation on Physical Training After Stroke. Stroke.

[31] Franceschini M., Ceravolo M.G., Agosti M., Cavallini P., Bonassi S., Dall’Armi V., Massucci M., Schifini F., Sale P. (2012). Clinical relevance of action observation in upper-limb stroke rehabilitation: A possible role in recovery of functional dexterity. A randomized clinical trial. Neurorehabilit. Neural Repair.

[32] Zhu J.-D., Cheng C.-H., Tseng Y.-J., Chou C.-C., Chen C.-C., Hsieh Y.-W., Liao Y.-H. (2019). Modulation of Motor Cortical Activities by Action Observation and Execution in Patients with Stroke: An MEG Study. Neural Plast..

[33] Rizzolatti G., Craighero L. (2004). THE MIRROR-NEURON SYSTEM. Annu. Rev. Neurosci..

[34] Grezes J., Decety J. (2001). Functional anatomy of execution, mental simulation, observation, and verb generation of actions: A meta-analysis. Human Brain Mapp..

[35] Di Pellegrino G., Fadiga L., Fogassi L., Gallese V., Rizzolatti G. (1992). Understanding motor events: A neurophysiological study. Exp. Brain Res..

[36] Gallese V., Fadiga L., Fogassi L., Rizzolatti G. (2009). Action recognition in the premotor cortex. Brain.

[37] Cattaneo L., Rizzolatti G. (2009). The Mirror Neuron System. Arch. Neurol..

[38] Chong T.T.-J., Cunnington R., Williams M.A., Kanwisher N., Mattingley J.B. (2008). fMRI Adaptation Reveals Mirror Neurons in Human Inferior Parietal Cortex. Curr. Biol..

[39] Buccino G., Binkofski F., Riggio L. (2004). The mirror neuron system and action recognition. Brain Lang..

[40] Zhang J.J.Q., Fong K.N.K., Welage N., Liu K.P.Y. (2018). The Activation of the Mirror Neuron System during Action Observation and Action Execution with Mirror Visual Feedback in Stroke: A Systematic Review. Neural Plast..

[41] Krams M., Rushworth M.F.S., Deiber M.-P., Frackowiak R.S.J., Passingham R.E. (1998). The preparation, execution and suppression of copied movements in the human brain. Exp. Brain Res..

[42] Frenkel-Toledo S., Liebermann D.G., Bentin S., Soroker N. (2016). Dysfunction of the Human Mirror Neuron System in Ideomotor Apraxia: Evidence from Mu Suppression. J. Cogn. Neurosci..

[43] Iacoboni M. (2009). Imitation, Empathy, and Mirror Neurons. Annu. Rev. Psychol..

[44] Chen W., Ye Q., Ji X., Zhang S., Yang X., Zhou Q., Cong F., Chen W., Zhang X., Zhang B. (2015). Mirror neuron system based therapy for aphasia rehabilitation. Front. Psychol..

[45] Perry A., Saunders S.N., Stiso J., Dewar C., Lubell J., Meling T.R., Solbakk A.-K., Endestad T., Knight R.T. (2017). Effects of prefrontal cortex damage on emotion understanding: EEG and behavioural evidence. Brain.

[46] Macuga K.L., Frey S.H. (2012). Neural representations involved in observed, imagined, and imitated actions are dissociable and hierarchically organized. NeuroImage.

[47] Watanabe R., Watanabe S., Kuruma H., Murakami Y., Seno A., Matsuda T. (2011). Neural activation during imitation of movements presented from four different perspectives: A functional magnetic resonance imaging study. Neurosci. Lett..

[48] Bekkering H., Wohlschlager A., Gattis M. (2000). Imitation of gestures in children is goal-directed. Q. J. Exp. Psychol. Sect. A.

[49] Koski L., Iacoboni M., Dubeau M.-C., Woods R.P., Mazziotta J.C., Kann O., Kovacs R., Heinemann U. (2003). Modulation of Cortical Activity During Different Imitative Behaviors. J. Neurophysiol..

[50] Miyamoto R., Kikuchi Y., Senoo A. (2008). Distinctive neural basis of anatomic imitation compared with specular imitation. J. J. Jpn. Acad. Health Sci..

[51] Mengotti P., Corradi-Dell’Acqua C., Rumiati R.I. (2012). Imitation components in the human brain: An fMRI study. NeuroImage.

[52] Aziz-Zadeh L., Koski L., Zaidel E., Mazziotta J., Iacoboni M. (2006). Lateralization of the Human Mirror Neuron System. J. Neurosci..

[53] Rizzolatti G., Sinigaglia C. (2010). The functional role of the parieto-frontal mirror circuit: Interpretations and misinterpretations. Nat. Rev. Neurosci..

[54] Saleh S., Yarossi M., Manuweera T., Adamovich S., Tunik E. (2017). Network interactions underlying mirror feedback in stroke: A dynamic causal modeling study. NeuroImage Clin..

[55] Avanzino L., Raffo A., Pelosin E., Ogliastro C., Marchese R., Ruggeri P., Abbruzzese G. (2014). Training based on mirror visual feedback influences transcallosal communication. Eur. J. Neurosci..

[56] Läppchen C., Ringer T., Blessin J., Schulz K., Seidel G., Lange R., Hamzei F. (2015). Daily iTBS worsens hand motor training—A combined TMS, fMRI and mirror training study. NeuroImage.

[57] Nojima I., Mima T., Koganemaru S., Thabit M.N., Fukuyama H., Kawamata T. (2012). Human Motor Plasticity Induced by Mirror Visual Feedback. J. Neurosci..

[58] Davis N.J., van Koningsbruggen M. (2013). “Non-invasive” brain stimulation is not non-invasive. Front. Syst. Neurosci..

[59] Rossi S., Antal A., Bestmann S., Bikson M., Brewer C., Brockmöller J., Carpenter L.L., Cincotta M., Chen R., Daskalakis J.D. (2020). Safety and recommendations for TMS use in healthy subjects and patient populations, with updates on training, ethical and regulatory issues: Expert Guidelines. Clin. Neurophysiol..

[60] Oldfield R. (1971). The assessment and analysis of handedness: The Edinburgh inventory. Neuropsychologia.

[61] Keel J.C., Smith M.J., Wassermann E.M. (2001). A safety screening questionnaire for transcranial magnetic stimulation. Clin. Neurophysiol..

[62] Jung P., Ziemann U. (2006). Differences of the ipsilateral silent period in small hand muscles. Muscle Nerve.

[63] Kuo Y.-L., Dubuc T., Boufadel D.F., Fisher B.E. (2017). Measuring ipsilateral silent period: Effects of muscle contraction levels and quantification methods. Brain Res..

[64] Baweja H.S., Patel B.K., Martinkewiz J.D., Vu J., Christou E.A. (2009). Removal of visual feedback alters muscle activity and reduces force variability during constant isometric contractions. Exp. Brain Res..

[65] Rossini P., Burke D., Chen R., Cohen L., Daskalakis Z., Di Iorio R., Di Lazzaro V., Ferreri F., Fitzgerald P., George M. (2015). Non-invasive electrical and magnetic stimulation of the brain, spinal cord, roots and peripheral nerves: Basic principles and procedures for routine clinical and research application. An updated report from an I.F.C.N. Committee. Clin. Neurophysiol..

[66] Chen R., Cros D., Curra A., Di Lazzaro V., Lefaucheur J.-P., Magistris M.R., Mills K., Rösler K.M., Triggs W.J., Ugawa Y. (2008). The clinical diagnostic utility of transcranial magnetic stimulation: Report of an IFCN committee. Clin. Neurophysiol..

[67] Ziemann U., Hallett M. (2001). Hemispheric asymmetry of ipsilateral motor cortex activation during unimanual motor tasks: Further evidence for motor dominance. Clin. Neurophysiol..

[68] Stinear C.M., Petoe M.A., Byblow W.D. (2015). Primary Motor Cortex Excitability During Recovery After Stroke: Implications for Neuromodulation. Brain Stimul..

[69] Davidson T., Tremblay F. (2013). Age and hemispheric differences in transcallosal inhibition between motor cortices: An ispsilateral silent period study. BMC Neurosci..

[70] Fleming M.K., Newham D.J. (2017). Reliability of Transcallosal Inhibition in Healthy Adults. Front. Hum. Neurosci..

[71] Takeuchi N., Tada T., Matsuo Y., Ikoma K. (2012). Low-Frequency Repetitive TMS Plus Anodal Transcranial DCS Prevents Transient Decline in Bimanual Movement Induced by Contralesional Inhibitory rTMS After Stroke. Neurorehabilit. Neural Repair.

[72] Kessler K., Biermann-Ruben K., Jonas M., Siebner H.R., Bäumer T., Münchau A., Schnitzler A. (2006). Investigating the human mirror neuron system by means of cortical synchronization during the imitation of biological movements. NeuroImage.

[73] Binder E., Dovern A., Hesse M.D., Ebke M., Karbe H., Saliger J., Fink G.R., Weiss-Blankenhorn P. (2017). Lesion evidence for a human mirror neuron system. Cortex.

[74] Gatti R., Rocca M.A., Fumagalli S., Cattrysse E., Kerckhofs E., Falini A., Filippi M. (2017). The effect of action observation/execution on mirror neuron system recruitment: An fMRI study in healthy individuals. Brain Imaging Behav..

[75] Iacoboni M., Hurley S., Chater N. (2005). Understanding Others: Imitation, Language, Empathy. Perspectives on Imitation: From Cognitive Neuroscience to Social Science.

[76] Franz E.A., Ford S., Werner S. (2007). Brain and cognitive processes of imitation in bimanual situations: Making inferences about mirror neuron systems. Brain Res..

[77] Wapner S., Cirillo L. (1968). Imitation of a model’s hand movements: Age changes in transposition of left-right relations. Child Dev..

[78] Pierpaoli C., Ferrante L., Manzoni T., Fabri M. (2014). Anatomical or mirror mode imitation? A behavioral approach. Arch Ital Biol.

[79] Hübers A., Orekhov Y., Ziemann U., Hbers A. (2008). Interhemispheric motor inhibition: Its role in controlling electromyographic mirror activity. Eur. J. Neurosci..

[80] Fling B.W., Seidler R.D. (2012). Task-dependent effects of interhemispheric inhibition on motor control. Behav. Brain Res..

[81] Pierpaoli C., Foschi N., Cagnetti C., Ferrante L., Manzoni T., Polonara G., Fabri M. (2018). Imitation Strategies in Callosotomized Patients. Arch. Ital. Biol..

[82] Netz J., Ziemann U., Hömberg V. (1995). Hemispheric asymmetry of transcallosal inhibition in man. Exp. Brain Res..

[83] Duque J., Murase N., Celnik P., Hummel F., Harris-Love M., Mazzocchio R., Olivier E., Cohen L.G. (2007). Intermanual Differences in Movement-related Interhemispheric Inhibition. J. Cogn. Neurosci..

[84] Moulton E., Galléa C., Kemlin C., Valabregue R., Maier M.A., Lindberg P., Rosso C. (2017). Cerebello-Cortical Differences in Effective Connectivity of the Dominant and Non-dominant Hand during a Visuomotor Paradigm of Grip Force Control. Front. Hum. Neurosci..

[85] Kirby K.M., Pillai S.R., Carmichael O.T., Van Gemmert A.W.A. (2019). Brain functional differences in visuo-motor task adaptation between dominant and non-dominant hand training. Exp. Brain Res..

[86] Malihe Moones T., Emami T., Hoseini S.M. (2017). The Effect of Initial Practice with Dominant and Non-Dominant Hand on Acquisition, Retention and Transfer of A Complex Motor Task. Biosci. Biotechnol. Res. Asia.

[87] Nelson A.J., Hoque T., Gunraj C., Ni Z., Chen R. (2009). Bi-directional interhemispheric inhibition during unimanual sustained contractions. BMC Neurosci..

[88] Grefkes C., Eickhoff S.B., Nowak D.A., Dafotakis M., Fink G.R. (2008). Dynamic intra- and interhemispheric interactions during unilateral and bilateral hand movements assessed with fMRI and DCM. NeuroImage.

[89] Casula E.P., Maiella M., Pellicciari M.C., Porrazzini F., D’Acunto A., Rocchi L., Koch G. (2020). Novel TMS-EEG indexes to investigate interhemispheric dynamics in humans. Clin. Neurophysiol..

[90] Casula E.P., Pellicciari M.C., Bonnì S., Spanò B., Ponzo V., Salsano I., Giulietti G., Cinnera A.M., Maiella M., Borghi I. (2020). Evidence for interhemispheric imbalance in stroke patients as revealed by combining transcranial magnetic stimulation and electroencephalography. Human Brain Mapp..

